# A novel and clinically useful dynamic conformal arc (DCA)‐based VMAT planning technique for lung SBRT

**DOI:** 10.1002/acm2.12878

**Published:** 2020-04-19

**Authors:** Damodar Pokhrel, Justin Visak, Lana Sanford

**Affiliations:** ^1^ Department of Radiation Medicine Medical Physics Graduate Program University of Kentucky Lexington KY USA; ^2^ Department of Radiation Medicine University of Kentucky Markey Cancer Center Lexington KY USA

**Keywords:** DCA‐based VMAT, FFF‐beam, lung SBRT, single dose

## Abstract

**Purpose:**

Volumetric modulated arc therapy (VMAT) is gaining popularity for stereotactic treatment of lung lesions for medically inoperable patients. Due to multiple beamlets in delivery of highly modulated VMAT plans, there are dose delivery uncertainties associated with small‐field dosimetry error and interplay effects with small lesions. We describe and compare a clinically useful dynamic conformal arc (DCA)‐based VMAT (*d*‐VMAT) technique for lung SBRT using flattening filter free (FFF) beams to minimize these effects.

**Materials and Methods:**

Ten solitary early‐stage I‐II non‐small‐cell lung cancer (NSCLC) patients were treated with a single dose of 30 Gy using 3–6 non‐coplanar VMAT arcs (clinical VMAT) with 6X‐FFF beams in our clinic. These clinically treated plans were re‐optimized using a novel *d*‐VMAT planning technique. For comparison, *d*‐VMAT plans were recalculated using DCA with user‐controlled field aperture shape before VMAT optimization. Identical beam geometry, dose calculation algorithm, grid size, and planning objectives were used. The clinical VMAT and *d*‐VMAT plans were compared via RTOG‐0915 protocol compliances for conformity, gradient indices, and dose to organs at risk (OAR). Additionally, treatment delivery efficiency and accuracy were recorded.

**Results:**

All plans met RTOG‐0915 requirements. Comparing with clinical VMAT, *d*‐VMAT plans gave similar target coverage with better target conformity, tighter radiosurgical dose distribution with lower gradient indices, and dose to OAR. Lower total number of monitor units and small beam modulation factor reduced beam‐on time by 1.75 min (*P* < 0.001), on average (maximum up to 2.52 min). Beam delivery accuracy was improved by 2%, on average (*P* < 0.05) and maximum up to 6% in some cases for *d*‐VMAT plans.

**Conclusion:**

This simple *d*‐VMAT technique provided excellent plan quality, reduced intermediate dose‐spillage, and dose to OAR while providing faster treatment delivery by significantly reducing beam‐on time. This novel treatment planning approach will improve patient compliance along with potentially reducing intrafraction motion error. Moreover, with less MLC modulation through the target, *d*‐VMAT could potentially minimize small‐field dosimetry errors and MLC interplay effects. If available, *d*‐VMAT planning approach is recommended for future clinical lung SBRT plan optimization.

## 1 INTRODUCTION

The use of stereotactic body radiation therapy (SBRT) has become a standard curative treatment for medically inoperable early‐staged non‐small‐cell lung cancer (NSCLC) patients providing a high cure rate and minimal treatment‐related toxicity.^1^, ^2^, ^3^, ^4^, ^5^ For the selected peripherally located NSCLC patients, single dose of lung SBRT has become a curative‐intent treatment option as shown by the randomized clinical trials.^6^, ^7^, ^8^, ^9^, ^10^, ^11^, ^12^, ^13^ Most recently, clinical use of flattening filter free (FFF) beams has been of interest in delivering lung SBRT treatments due to dosimetric advantages compared to a flattened beam.^14^, ^15^, ^16^, ^17^, ^18^ FFF beams can significantly reduce beam‐on time due to their higher dose rates, resulting in better patient compliance, potentially reducing dose delivery uncertainty due to less intrafraction motion error and reduction in out‐of‐field dose with less head scatter and electron contamination.^14^, ^15^, ^16^

A single‐large dose of 30 Gy in one fraction lung SBRT treatment is an extreme form of hypofractionation dosing schemata used in our clinic for extracranial lesions where the dose calculation accuracy could potentially suffer by tumor size, tumor location, and the presence of tissue heterogeneity in the lung. Utilizing volumetric modulated arc therapy (VMAT) with FFF‐beams^17^, ^18^ resulted in better tumor dose coverage and faster treatment delivery of complex lung SBRT treatments compared to historically used plans with 8–15 non‐coplanar fixed fields or several coplanar DCA fields with flattened beams.^19^, ^20^, ^21^, ^22^ Similar results were observed when compared to linac‐based intensity modulated radiation therapy (IMRT), VMAT plans, helical TomoTherapy, or optimized robotic CyberKnife plans (showing significant increases in SBRT treatment times).^23^, ^24^, ^25^, ^26^ However, for a single dose of lung SBRT treatments, highly modulated IMRT/VMAT plans are susceptible to delivery uncertainties due to small‐field dosimetry error^27^ and interplay effects^28^ due to MLC modulation of multiple beamlets through the target as a function of lung tumor motion and tissue heterogeneities.

To minimize these effects, recently, Varian Eclipse treatment planning system (TPS, Varian Medical Systems, Palo Alto CA, Version 15.1 and beyond)^29^ has implemented a new multileaf collimator (MLC) optimization algorithm, called Photon Optimizer (PO). PO offers a new MLC aperture shaper controller. With this new feature, users can control the field aperture shape and create a 3D plan using dynamic conformal arc (DCA) therapy before VMAT optimization. Although a few investigators have studied the clinical use of PO‐MLC algorithm for VMAT lung SBRT plan optimization,^30^, ^31^ the dosimetric impact and treatment delivery complexity of this planning approach with a FFF beam in the treatment of single high dose (30 Gy in 1 fraction) using non‐coplanar VMAT lung SBRT plan has not yet been reported.

As part of SBRT commissioning of Eclipse TPS (Version 15.6), it is important to stress the importance of investigating new planning features to provide the highest quality and most accurate plan. Dose to radiosensitive non‐target OAR is a major concern in VMAT lung SBRT treatments,^32^, ^33^ specifically while delivering a single‐large fraction dose as described here. Herein, we have retrospectively evaluated 10 consecutive early‐stage NSCLC patient's plans who underwent a single dose of VMAT lung SBRT treatment in our clinic. For comparison, the clinical VMAT plans were re‐optimized using a DCA‐based VMAT (*d*‐VMAT) planning approach with identical beam geometry, dose calculation algorithm, grid size, planning objectives, and parameters. The *d*‐VMAT plans utilized DCA‐based dose with the highest strength of field aperture‐shape control priority before VMAT optimization; therefore, less beam modulation through the target is expected. The original clinical VMAT plans and re‐optimized *d*‐VMAT plans were compared via lung SBRT protocol compliance criteria for the target conformity, intermediate dose‐spillage, and dose to OAR per RTOG requirements.^6^ Furthermore, treatment delivery efficiency and accuracy were reported.

## 2 MATERIALS AND METHODS

### 2.A Patient characteristics

After obtaining an institutional review board (IRB) approval from our institution, 10 consecutive Stage I‐II NSCLC patients with peripherally located tumors who underwent a single dose of lung SBRT treatments (30 Gy) were included in this study.

### 2.B Imaging and target definition

All patients were immobilized using Body Pro‐Lok^TM^ platform (CIVCO system, Orange City, IA) in the supine position with their arms above their head using an armrest. The free‐breathing planning 3D‐CT scan was acquired on a GE Lightspeed 16 slice CT scanner (General Electric Medical Systems, Waukesha, WI) with 512 × 512 pixels at 2.5 mm slice thickness in the axial helical mode. Following the 3D‐CT scan, these patients underwent a respiration‐correlated 4D‐CT scan using the Varian RPM System (version 1.7) in the same position. The 4D‐CT images were reconstructed in 10 equally spaced phase bins using an Advantage 4D Workstation (GE Medical Systems, San Francisco, CA), where the maximum intensity projection (MIP) images were generated. The regular 3D‐CT and the MIP images were imported into Eclipse TPS (Version 15.6, Varian Medical Systems, Palo Alto, CA) and co‐registered for target delineation. An internal target volume (ITV) was created using the 4D‐MIP co‐registered with planning 3D‐CT images. Planning target volume (PTV) was generated by adding a 5 mm isotropic margin around the ITV per RTOG‐0915 recommendation.^6^ The relevant critical structures included bilateral lungs excluding the ITV (healthy lung), spinal cord, ribs, heart, trachea/bronchus, esophagus, and skin.

The tumor characteristics are summarized in Table 1 including tumor size and location. The average ITV derived from the 4D‐CT scan was 6.3 ± 4.9 cc (range: 0.5–15.2 cc). The mean PTV was 23.3 ± 13.6 cc (range: 6.5–43.8 cc), corresponding to an average tumor diameter of 3.4 ± 0.7 cm (range: 2.3–4.3 cm).

**Table 1:** Characteristics of lung SBRT patients included in this study. Prescription was 30 Gy in 1 fraction.

Patient no.	Tumor location	ITV (cc)	PTV (cc)	PTV diameter, *d* (cm)	Normal lung volume (cc)
1	Left upper lobe	2.1	14.8	3.0	3708
2	Right lower lobe	15.2	37.5	4.1	4088
3	Right upper lobe	3.0	12.8	2.9	3493
4	Left upper lobe	4.2	15.2	3.0	4218
5	Right upper lobe	12.2	37.6	4.1	5100
6	Left upper lobe	8.5	35.8	4.0	4456
7	Right upper lobe	3.1	14.4	3.0	5981
8	Right lower lobe	10	43.8	4.3	5323
9	Left upper lobe	3.9	14.3	3.0	4584
10	Left upper lobe	0.5	6.5	2.3	6027

### 2.C Clinical VMAT plans and treatment delivery

For the 10 consecutive patients, clinically optimal VMAT‐SBRT plans were generated in Eclipse TPS using 3–6 (mean, 4) partial non‐coplanar arcs (with ± 5–10° couch kicks) for a Truebeam Linac (Varian Palo Alto, CA) consisting of standard millennium 120 MLC and 6 MV‐FFF (1400 MU/min) beam. The isocenter position was set to the geometric center of the PTV. These partial non‐coplanar arcs had an arc length of approximately 200–220°. Collimator angles (between 30 and 135°) were manually optimized to reduce the MLC tongue‐and‐groove dose leakage throughout the arc rotation on a per‐patient basis. Additionally, the jaw‐tracking option was used during plan optimization to further minimize out‐of‐field dose leakage. The prescription dose was 30 Gy in 1 fraction to the PTV while covering at least 95% of the PTV with prescription dose and ensuring that all hot spots (between 120 and 130%) fall within the ITV. All clinical treatment plans were calculated with the advanced AcurosXB (Varian Eclipse TPS, Version 15.6) dose calculation algorithm^34^, ^35^, ^36^, ^37^ on the planning 3D‐CT images with heterogeneity corrections with 1.25 × 1.25 × 1.25 mm^3^ CGS and the PO‐MLC algorithm. In these clinical plans, low priority of MLC aperture shape controller was used. The dose to medium reporting mode was applied, and the planning objectives followed the RTOG‐0915 requirements (Arm 1).^6^

Before delivering each VMAT‐SBRT plan, a daily quality assurance (QA) check on kilovoltage to megavoltage imaging isocenter coincidence was performed, including IsoCal measurement for the precise and accurate target localization. IsoCal localization accuracy for our Truebeam Linac was < 0.5 mm. All the QA procedures, including patient‐specific QA, were in compliance for SBRT treatment delivery.^5^ On the treatment day, patient set up prior to single‐dose lung SBRT was performed using SBRT/IGRT protocol; ^5^, ^6^ by co‐registering the pretreatment conebeam CT with the planning CT scan. Image registration was performed, automatically, based on a bony landmark region of interest, followed by manual refining performed by the treating physician. The patient positon was re‐positioned using 6 degrees of freedom (6‐DoF) couch corrections according to the results of tumor soft tissue registration prior to treatment delivery. The 6‐DoF couch correction parameters were within the limits of our departmental lung SBRT protocol guidelines for all patients. The patient set up, tumor matching, and treatment delivery were monitored and verified by the treating physician and physicist.

### 2.D Re‐optimized *d*‐VMAT plans

**Fig. 1:**
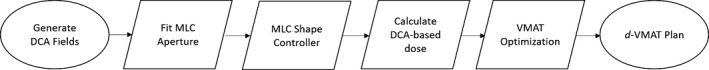
Proposed workflow of *d*‐VMAT treatment planning technique for a single dose of lung SBRT.

For comparison, the standard clinical VMAT plans for all patients were retrospectively re‐planned (in Eclipse v15.6) using a new feature (MLC aperture controller) with DCA‐based dose calculation followed by VMAT optimization (*d*‐VMAT). See Fig. 1 for the proposed workflow of this novel approach. For the *d*‐VMAT plans (with identical beam geometry), the first 2 mm of the MLC aperture around the PTV is automatically generated and maintained dynamically around the target during arc rotation. The MLC was observed to dynamically conform to the beam's‐eye‐view (BEV) projections of the PTV for each arc. Second, very high priority in the MLC aperture shape controller was selected (a new feature in Eclipse v15.6, PO algorithm in calculation models) and proceeded to calculate a DCA‐based 3D dose distribution. This 3D dose distribution was used as a dose plan for VMAT optimization. This was followed by VMAT optimization with identical planning objectives, dose calculation algorithm, grid size, and convergence mode identical to the original clinical VMAT plan, including the normal tissue objectives (NTO) parameters and ring structures.

### 2.E Plan evaluation and statistical analysis

The original clinical VMAT and re‐optimized *d*‐VMAT plans were compared using RTOG‐0915 protocol compliance criteria for target conformity (CI), heterogeneity index (HI), gradient indices (GI), and dose to OAR. Additionally, delivery efficiency and accuracy were recorded. The DVHs of all treatment plans were evaluated following RTOG‐0915 high and intermediate dose‐spillage dose parameters: ^6^

- Conformity index, CI: ratio of prescription isodose volume to the PTV. CI less than 1.2 is desirable; CI = 1.2–1.5 is acceptable with minor deviations.
- Gradient index, GI: ratio of 50% prescription isodose volume to the PTV. GI has to be smaller than 3–6, depending on the PTV.
- Maximum dose at any point 2 cm away from the PTV margin in any direction, D_2cm_: D_2cm_ has to be smaller than 50–70%, depending on the PTV size.
- Percentage of normal lung receiving dose equal to 20 Gy or more, V_20_: Per protocol, V_20_ should be less than 10%, V_20_ less than 15% is acceptable with minor deviations.
- Heterogeneity index, HI: HI = Dmax/prescribed dose was used to evaluate the dose heterogeneity within the PTV.
- Gradient distance, GD: GD is the average distance from 100% prescribed dose to 50% prescribed dose, which indicates how sharp the dose falls off. The GD is used to evaluate dose sparing to normal lung volume. The smaller the value of GD, the faster the dose fall‐off around the target.
- Total number of monitor units (MU).
- Modulation factor, MF: ratio of total number of MU to the prescription dose in cGy.
- Beam‐on time, BOT: BOT was recorded during VMAT QA phantom measurement at the machine for both plans.

Furthermore, all clinical VMAT and *d*‐VMAT plans were evaluated for the relative volume of normal lung receiving 10 Gy, dose to the spinal cord (maximum and 0.35 cc), heart (maximum and 15 cc), and esophagus (maximum and 5 cc). Since these tumors were peripherally located, the doses to ribs (maximum and 1 cc) and skin (maximum and 10 cc) were also documented. The mean and standard deviation for each dose metric were compared using two‐tailed students t‐tests (using an upper bound *P* value of < 0.05, being statistically significant) for the clinical VMAT vs *d*‐VMAT plans for all dosimetric parameters, target coverage, dose tolerances to OAR, and treatment delivery parameters. Dose limits for maximum doses to spinal cord <14.0 Gy, heart <22.0 Gy, esophagus <15.4 Gy, maximum point dose and dose to 1 cc of ribs, <30.0 Gy and <22.0 Gy, maximum point dose and 10 cc of skin <26.0 Gy and <23.0 Gy were used per single‐dose lung SBRT protocol (RTOG‐0915, Arm 1) requirements, respectively.

## 3 RESULTS

### 3.A Target coverage

All plans were acceptable per RTOG‐0915 requirements. Compared to clinical VMAT plans, *d*‐VMAT plans showed similar tumor conformity, dose heterogeneity, and target coverage; all exhibited no statistical significance. However, *d*‐VMAT plans show better gradient indices as demonstrated by the values of intermediate dose‐spillage (GI, D_2cm_ and GD, see Table 2), systematically lower for all patient's plans.

**Table 2:** Evaluation of target coverage for all 10 lung SBRT patients for both plans.

Target volume	Parameters	Clinical VMAT	*d*‐VMAT	*P* value
PTV	CI	1.04 ± 0.08 (0.98–1.24)	1.02 ± 0.03 (0.99–1.05)	*P = 0.102*
	HI	1.24 ± 0.04 (1.18–1.29)	1.22 ± 0.04 (1.16 –1.31)	*P = 0.248*
	GI	4.9 ± 0.9 (3.7–6.5)	4.6 ± 0.7 (3.7–5.7)	***P = 0.009***
	D_2cm_ (%)	51.1 ± 3.5 (45.7–57.6)	48.6 ± 2.6 (45.3–53.7)	***P = 0.002***
	GD (cm)	1.13 ± 0.13 (0.94–1.34)	1.10 ± 0.13 (0.93–1.27)	***P = 0.002***
ITV	D_min_ (Gy)	31.69 ± 1.12 (29.37–33.7)	31.90 ± 1.43 (29.09–34.13)	*P = 0.267*
	D_max_ (Gy)	37.03 ± 1.24 (35.28–38.78)	36.90 ± 1.46 (34.90–39.25)	*P = 0.604*
	D_mean_ (Gy)	34.42 ± 0.97 (33.06–35.46)	34.63 ± 0.89 (33.33–35.51)	*P = 0.094*

Prescription was 30 Gy in 1 fraction. Mean ± SD (range) was reported. SD = standard deviation. Statistically significant *P* values are highlighted in bold.

**Fig. 2:**
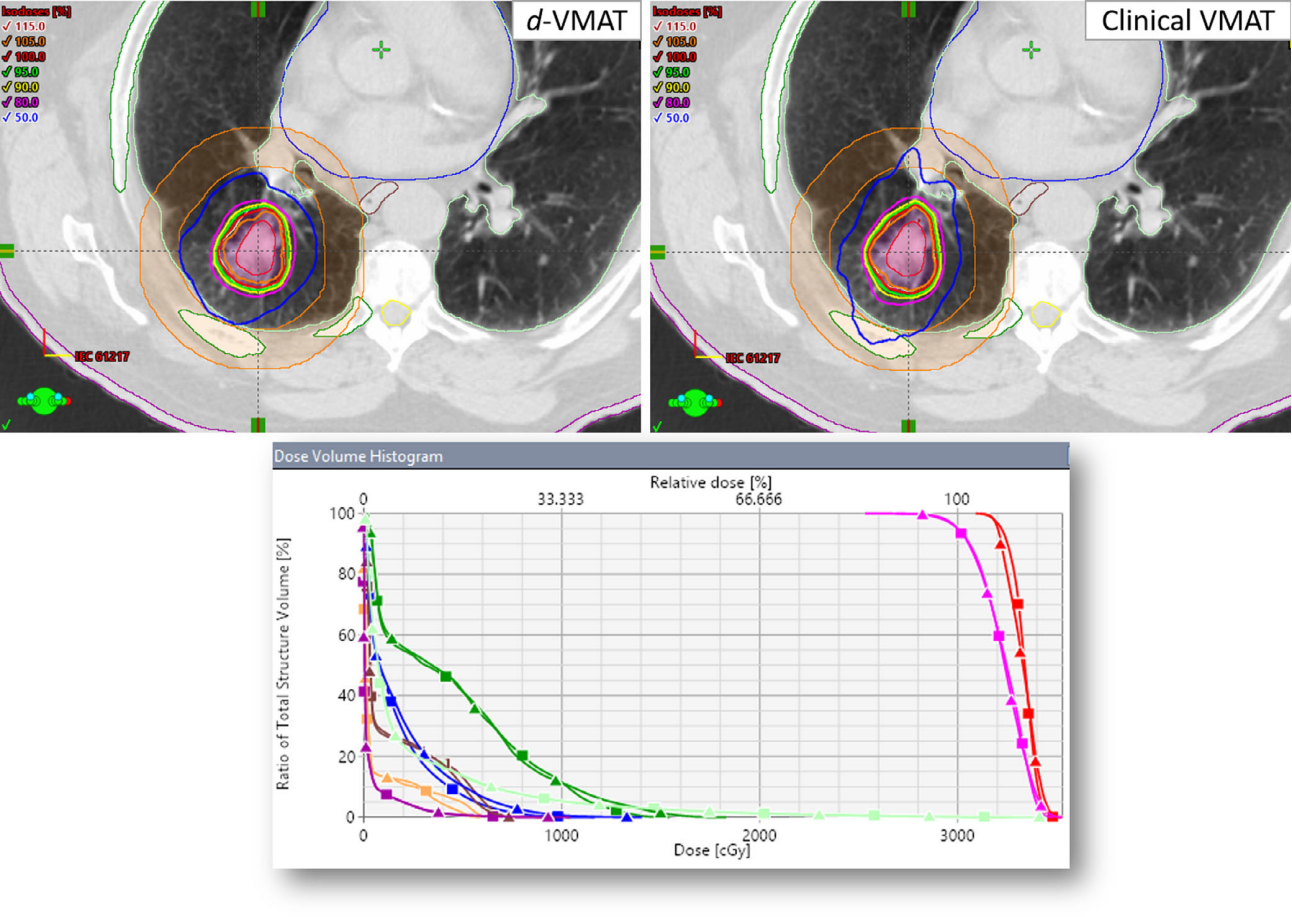
Comparison of clinical VMAT vs a *d*‐VMAT plan for the example case #6 described above. The upper panel shows radiosurgical isodose distributions for the *d*‐VMAT (left) and clinical VMAT plan (right) — crosshair shows the isocenter location. Better values of CI, HI, GI, D2cm, GD, and V_20Gy_ were obtained with *d*‐VMAT plan. A few critical structures shown were ribs, cord, normal lung, heart, esophagus as well as D_2cm_ ring (orange color contour). The lower panel shows the DVH comparison for the both plans. Triangle shows the clinical VMAT and square shows the *d*‐VMAT plan (red, ITV; Pink, PTV; green, ribs; light blue, normal lung; brown, esophagus; blue, heart and dark magenta, skin). Identical target coverage and similar OAR sparing were achieved with *d*‐VMAT plan; but, it gave better gradient indices, a shorter treatment time, and perhaps more accurate treatment delivery.

No major dosimetric differences in terms of dose to OAR were observed. Both plans achieved the RTOG‐0915 protocol compliance criteria and were clinically acceptable for stereotactic treatment of lung lesions. Figure 2 shows an example case of radiosurgical dose distribution in the axial view through the isocenter plane for an example lung SBRT patient planned with clinical VMAT (top right panel) and *d*‐VMAT (top left panel). However, clinically desirable tighter 50% isodose distribution was obtained with *d*‐VMAT (see blue isodose lines) compared to the clinical VMAT plan. DVH parameters (bottom panel) are shown for the target coverage and dose to OAR for clinical VMAT vs *d*‐VMAT plan, suggesting dosimetrically comparable plans. The PTV size was 35.8 cc (4.0 cm diameter). This is a relatively large tumor size in this cohort and located in the left upper lobe. In this case, the CI, HI, GI, D_2cm_, GD, and normal lung V_20 Gy_ were 1.00 vs 1.04, 1.16 vs 1.18, 4.3 vs 4.6, 50.4% vs 52.4%, 1.27 cm vs 1.34 cm, and 1.7% vs 2.0%, *d*‐VMAT vs clinical VMAT plan, respectively — all parameters favoring the *d*‐VMAT plan.

### 3.B OAR Sparing

The dosimetric differences (mean and standard deviation) between clinical VMAT and *d*‐VMAT plans for the OAR (spinal cord, heart, esophagus, trachea/bronchus, ribs, skin, and normal lung) are listed in Table 3. Although statistically insignificant differences (*P* value> 0.05) were found for the most of the evaluated dosimetric parameters (excluding dose to ribs and normal lung V20_Gy_), there is a clear trend of slightly decreased dose to OAR with *d*‐VMAT plans (see highlighted *P* values). Despite the dose to ribs and V_20 Gy_ presenting average absolute differences of about 1.0 Gy and 0.1%, these values are well below RTOG guidelines and therefore are not expected to be clinically significant.

**Table 3:** Evaluation of dose to OAR and treatment delivery efficiency for all 10 lung SBRS patients for both plans.

Dose to OAR	Parameters	Clinical VMAT	*d*‐VMAT	*P* value
Spinal cord (Gy)	D_max_	5.1 ± 2.0 (1.0–7.9)	4.9 ± 2.1(1.1–8.1)	*P = 0.208*
	D_0.35 cc_	4.6 ± 1.8 (0.9–7.2)	4.5 ± 1.9 (1.1–7.4)	*P = 0.411*
Heart/pericardium (Gy)	D_max_	5.9 ± 4.9 (0.4–13.7)	5.3 ± 4.3 (0.4–11.2)	*P = 0.084*
	D_15 cc_	3.6 ± 3.2 (0.2–9.0)	3.4 ± 2.9 (0.2–7.7)	*P = 0.187*
Esophagus (Gy)	D_max_	5.8 ± 1.9 (2.3–7.4)	5.6 ± 1.9 (2.2–7.7)	*P = 0.209*
	D_5 cc_	2.3 ± 1.3 (0.3–4.8)	2.3 ± 1.4 (0.2–5.2)	*P = 0.119*
Trachea/bronchus (Gy)	D_max_	9.7 ± 5.3 (3.2–18.9)	8.8 ± 4.5 (2.9–16.5)	*P = 0.061*
	D_4cc_	3.4 ± 2.2 (0.1–7.6)	2.8 ± 2.1 (0.3–6.8)	*P = 0.157*
Skin (Gy)	D_max_	9.4 ± 2.3 (6.3–13.9)	9.0 ± 1.8 (5.8–12.3)	*P = 0.199*
	D_10 cc_	5.6 ± 1.7 (3.9–7.6)	5.4 ± 1.6 (4.1–8.3)	*P = 0.115*
Ribs (Gy)	D_max_	15.5 ± 4.0 (11.3–22.7)	14.1 ± 3.0 (10.1–18.8)	***P = 0.006***
	D_1cc_	13.7 ± 3.3 (9.8–19.3)	12.7 ± 2.7 (9.4–16.6)	***P = 0.009***
Normal lung (%)	V_20 Gy_	1.2 ± 0.5 (0.3–2.0)	1.1 ± 0.5 (0.3–1.9)	***P = 0.008***
	V_10 Gy_	4.1 ± 1.7 (1.5–6.4)	3.6 ± 1.6 (1.6–5.9)	*P = 0.209*

Prescription was 30 Gy in 1 fraction. Mean ± SD (range) was reported. SD = standard deviation. Statistically significant *p* values are highlighted in bold.

### 3.C Treatment delivery efficiency and accuracy

**Fig. 3:**
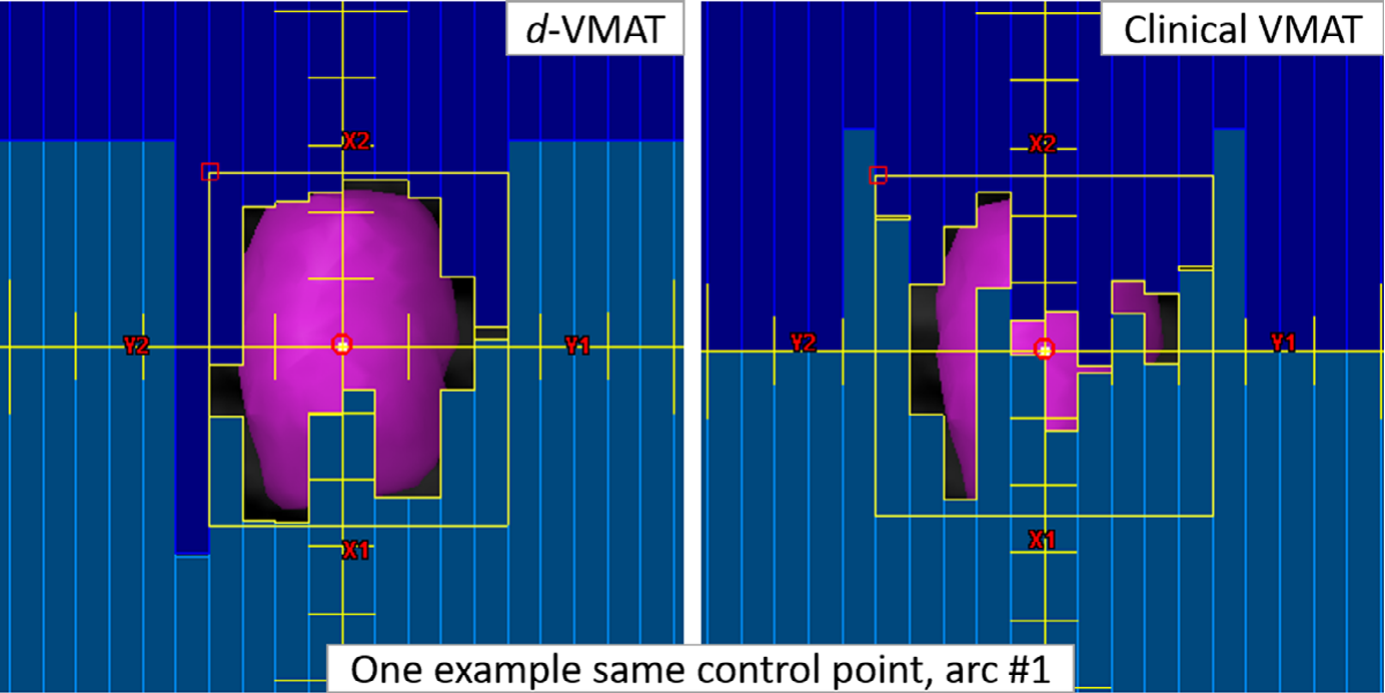
Comparison of a selected MLC control point (one control point for arc #1 on each plan) between the *d*‐VMAT and clinical VMAT plan (same patient shown in Fig. 1). The *d*‐VMAT MLC pattern (left panel) conforms to the PTV (pink) with less modulation while the majority of the PTV is under the MLC block, due to highly modulated MLC pattern, in the clinical VMAT plan (see, right panel). Although both plans provided similar target coverage and dose to OAR, *d*‐VMAT plan provided better gradient indices and delivered treatment much faster and more accurate due to less MLC modulation across the PTV.

The improvement of treatment delivery efficiency and accuracy is directly associated with Eclipse’s new feature of adjustable aperture shape control priority, which allows for DCA‐based dose calculation before VMAT optimization (Version 15.6). With this new feature, less beam modulation through the PTV was obtained and shown here for the same control point (in Fig. 3). In addition to the uncertainty of modeling small‐field dosimetry, there is a potential concern that the interplay effects between the very high dynamic MLC modulation and tumor motion can degrade the delivery accuracy compared to the calculated values based on static plans. This study does not quantify the variation of the delivered dose due to the tumor motion when calculating the dose distributions. However, with *d*‐VMAT less beam modulation to the projection of the target at each gantry angle (see Fig. 3) and similar MLC patterns were obtained for other control points, thus potentially minimizing MLC modulation and reducing the interplay effects. Therefore, the main benefit of the *d*‐VMAT plan is the reduced total MU and beam‐on time required to deliver the same prescription dose.

**Fig. 4:**
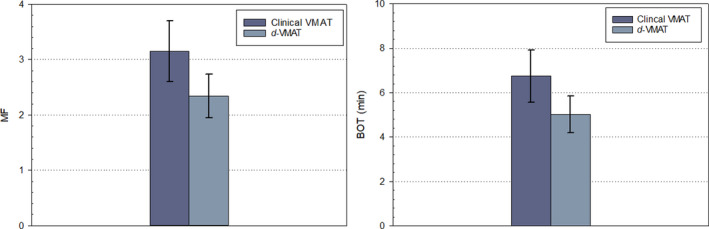
Left panel: MF for clinical VMAT vs *d*‐VMAT plans for all 10 patients treated with a single dose of 30 Gy to lung lesions. Mean values of MF for clinical VMAT and d‐VMAT plans were 3.15 ± 0.55 (range: 2.0–3.88) and 2.34 ± 0.39 (range: 1.55–2.75), respectively. Right panel: The corresponding BOT for clinical VMAT vs *d*‐VMAT plans. Mean values of BOT for clinical VMAT vs *d*‐VMAT plans were 6.74 ± 1.18 min (range: 4.29–8.32 min) and 5.02 ± 0.83 min (range: 3.33–5.89 min) respectively, with *d*‐VMAT plans significantly improving the beam‐on time.

Dose delivery efficiency was accessed by comparing total number of MU and estimated beam‐on time while delivering QA plans at the machine. Compared to clinical VMAT plans, *d*‐VMAT plans show smaller number of total MU and less beam modulation. Mean values of total MU and MF were 9440 and 3.2 for clinical VMAT plans vs 7030 and 2.34 for *d*‐VMAT plans. The MF and the beam‐on time for clinical VMAT vs *d*‐VMAT plans is shown in Fig. 4. For the given *d*‐VMAT plans, the total number of MU was reduced significantly (by a factor of 1.35, on average, and systematically lower for all patients) while using DCA‐based dose before VMAT plan optimization, suggesting that the *d*‐VMAT plan had smaller MF (*P* < 0.001). Because of this, the average beam‐on time for *d*‐VMAT plan was 1.75 min less (*P* < 0.001) (maximum up to 2.52 min) than clinical VMAT plan (mean value, 6.74 min) due to less beam modulation through the target. With *d*‐VMAT plan, single dose of 30 Gy to lung lesion can be delivered in 5 min. The lower beam‐on time will reduce the time the patient is on the table, thus improving patient comfort and potentially reducing errors due to intra‐fraction tumor motion, as well.

Dose delivery accuracy was accessed by delivering both plans at Truebeam Linac in the QA mode using the ArcCHECK device that was implemented in our clinic (Sun Nuclear Corp., Melbourne, FL, Version 8.3) with the center cylindrical insert in place. The dose delivery accuracy of these clinical VMAT plans, and the corresponding *d*‐VMAT plans were 91.0 ± 5.5% and 93.1 ± 4.8%, on average, respectively, with 2%/2 mm global gamma passing rate criteria. In some cases (for small lesions), dose delivery accuracy was improved by up to 6% with *d*‐VMAT plans, suggesting that significant dose deviation (*P* < 0.05) can be seen with highly modulated clinical VMAT plans compared to *d*‐VMAT plans.

## 4 DISCUSSION

A novel and clinically useful lung SBRT planning approach via DCA‐based dose followed by VMAT optimization is presented per RTOG‐0915 compliance for rapid delivery of a single dose of 30 Gy to lung lesions. The new *d*‐VMAT‐SBRT plans were highly conformal and achieved similar target coverage (see Table 2) compared to clinical VMAT plans. For all patients, the *d*‐VMAT plans provided similar or better OAR (spinal cord, heart, esophagus, trachea/bronchus, ribs, and skin, see Table 3) sparing and were well below protocol dose requirements. The *d*‐VMAT plans required less total number of MU to deliver the same total prescribed dose due to less beam modulation across the target. Therefore, the beam‐on time was reduced significantly (average beam‐on time 5.02 min) demonstrating the efficiency of *d*‐VMAT plans for a single dose of lung SBRT treatments in this group of patients. With *d*‐VMAT plans, the beam‐on time can be reduced by 35–40% compared with clinical VMAT (average BOT 6.74 min). Furthermore, the treatment delivery accuracy was improved significantly (see Table 4) with measurements analyzed at 2%/2 mm gamma passing criteria.

**Table 4:** Comparison of average values of treatment delivery parameters (and range) between clinical VMAT and re‐optimized *d*‐VMAT plans for all 10 lung SBRT patients. Mean ± SD (range) was reported. SD = standard deviation.

Beam delivery parameters	Clinical VMAT	*d*‐VMAT	*P* value
Total monitor units (MU)	9440 ± 1653 (6000–11641)	7030 ± 1168 (4660–8245)	***P < 0.001***
Modulation factor (MF)	3.15 ± 0.55 (2.0–3.88)	2.34 ± 0.39 (1.55–2.75)	***P < 0.001***
Beam‐on time (min)	6.74 ± 1.18 (4.29–8.32)	5.02 ± 0.83 (3.33–5.89)	***P < 0.001***
2%/2 mm, γ‐pass rate (%)	91.0 ± 5.5 (86.0–98.6)	93.1 ± 4.8 (86.0–99.3)	***P < 0.05***

Statistically significant *P* values are highlighted in bold.

For the conformal treatment delivery, a study by Dong et al^38^ from UCLA compared 4π plans with 7–9 static‐beam IMRT plans and VMAT plans prescribed to 50 Gy in four fractions for 12 centrally located lung cancer patients. The 4π algorithm used up to 30 optimized coplanar/non‐coplanar fields. In their study, it was concluded that compared to IMRT and VMAT, the 4π plans gave significantly and consistently better target coverage and critical OAR sparing. However, the 4π treatment delivery time was not reported. We believe that delivering 30 c/n‐coplanar fields to treat lung SBRT patients would be clinically impractical for current Linac/clinic workflows. In contrast, utilizing our *d*‐VMAT approach with 6MV‐FFF beam can deliver quicker (within a few minutes) and effective curative single‐dose SBRT treatments for selected early‐staged NSCLC patients.

Potential concerns of changing respiratory motion patterns between the planning CT simulation and the time of treatment have been studied in the past by many investigators.^39^, ^40^, ^41^, ^42^ It has been reported in the literature that there were only small changes (within ± 3 mm) due to intrafractional and interfractional motion in lung SBRT treatments. In addition, the mean patient set up time from tumor localization to the end of treatment cone beam CT scan was about 40 min.^41^ It was recommended that a symmetrical 5 mm PTV margin around the ITV was adequate to address these potential set up errors. Furthermore, the interplay effect between the MLC modulation and gantry rotation as a function of tumor motion could introduce dose blurring on highly modulated VMAT plans, which can be of another concern for a single high dose of lung SBRT treatments.^28^ In our study, the average beam‐on time was 5.02 min for single‐fraction lung SBRT treatment with *d*‐VMAT and 6X‐FFF beam, decreasing the variation in dose delivery due to coughing or pain and making geographic miss less likely by improving the patient stability on the table. Moreover, better delivery accuracy of *d*‐VMAT plans can improve the major concerns of small‐filed dosimetry errors and MLC interplay effects that persist with highly modulated VMAT plans demonstrated by a higher QA pass rates of our *d*‐VMAT plans delivered in QA phantom measurement.

Other possible fears for lung SBRT treatments are low/intermediate dose‐spillage in the chest wall and ribs; ^43^, ^44^, ^45^ normal lung (V_20Gy_, V_10Gy_, and V_5Gy_),^33^, ^46^ and dose to skin.^47^ For instance, Pettersson et al^43^ studied large cohort of 68 NSCLC patients treated 45 Gy in three fractions of lung SBRT. Among the 33 patients with complete clinical and radiographic follow‐up exceeding 15 months, 13 rib fractures were found in seven patients. In their study, the logistic dose–response curve exhibited that the risk of radiation‐induced rib fractures following lung SBRT treatments was related to the dose to 2 cc of the rib. For a median follow‐up of 29 months, they showed that the 2 cc of rib receiving total 27.3 Gy in three fractions had a 5% chance of rib fracture. In the current study, our *d*‐VMAT plans provided sharp dose fall off around the target and lower dose tolerances to all OAR (including lower dose to rib, lung, and skin, see table 3) compared to clinical VMAT plans and all OAR dose metrics were well below the RTOG criteria. Therefore, we do not anticipate any acute or late toxicity. However, clinical follow‐up of tumor local‐control and treatment‐related toxicities of these patients is necessary.

In summary, each *d*‐VMAT plans were carefully evaluated using the dosimetric and treatment delivery parameters (including QA results for 2%/2 mm gamma criteria) listed in Tables 2, 3, 4. All parameters were deemed acceptable for both *d*‐VMAT and clinical VMAT plans per SBRT protocol requirements suggesting that *d*‐VMAT plans are dosimetrically superior to clinical VMAT plans. With *d*‐VMAT, faster treatment delivery is anticipated, potentially benefiting patients who cannot lie flat in the treatment position for longer time and reducing intrafraction motion error. Additionally, *d*‐VMAT minimizes the major concerns over the accuracy of the dose calculation and delivery errors for small fields (beamlets) in areas of tissue interfaces. Moreover, *d*‐VMAT was observed to minimize the MLC interplay effect as demonstrated with higher pass rates of QA on phantom measurement (see Table 4). The *d*‐VMAT planning can be easily adopted to any other disease sites (including 3–5 fractions lung SBRT) such as stereotactic treatment of brain or any abdominal/pelvis lesions such as liver, pancreas, or adrenal glands SBRT. Due to decreased total number of MU/treatment and smaller beam‐on time with *d*‐VMAT planning approach, deep inspiration breath‐hold lung SBRT treatments may be of value in future investigations. Moreover, the potential use of MLC shape controller strength in *d*‐VMAT planning approach for highly irregular large targets that overlapped with adjacent OAR will be further investigated.

## 5 CONCLUSION

A simple, yet clinically useful *d*‐VMAT planning technique was presented for a single dose of lung SBRT treatments. The *d*‐VMAT provided excellent plan quality with reduced intermediate dose‐spillage, reduced dose to normal lung, and other OAR (including rib) and provided faster treatment delivery by significantly reducing the beam‐on time when compared to clinical VMAT lung SBRT plans. Furthermore, due to less MLC modulation over the target*, d*‐VMAT minimized small‐field dosimetry errors and MLC interplay effects and provided better QA pass rates. If available, *d*‐VMAT planning method is recommended for future clinical lung SBRT plan optimization as it provides a superior plan quality with minimal changes to planning workflow.

## CONFLICT OF INTEREST

None.
